# B7-H1 and B7-H3 are independent predictors of poor prognosis in patients with non-small cell lung cancer

**DOI:** 10.18632/oncotarget.3097

**Published:** 2014-12-31

**Authors:** Yixiang Mao, Wei Li, Kai Chen, Yufeng Xie, Qiang Liu, Min Yao, Weiming Duan, Xiumin Zhou, Rongrui Liang, Min Tao

**Affiliations:** ^1^ Department of Oncology, the First Affiliated Hospital of Soochow University, Suzhou, China; ^2^ Department of Pathology, Renji Hospital, Shanghai Jiaotong University School of Medicine, Shanghai, China; ^3^ Department of Pathology, Punan Hospital, Shanghai, China; ^4^ Jiangsu Institute of Clinical Immunology, Suzhou, China

**Keywords:** B7-H1, B7-H3, non-small cell lung cancer, prognosis

## Abstract

B7-H1 and B7-H3, two members of the B7 family that are thought to regulate T-cell activation, are expressed in human non-small cell lung cancer (NSCLC). However, their prognostic significance is poorly understood. In the present study we reported that B7-H1 and B7-H3 were expressed in 96/128 (72.7%) and 89/128 (69.5%) samples, respectively. B7-H1 and B7-H3 expression and the number of infiltrating T-cell intracellular antigen-1+ and interferon-γ+ cells in NSCLC tissues were significantly higher than those in the adjacent tissues (p<0.01). High B7-H1 or B7-H3 expression was associated with lymph node metastasis and TNM stage (p<0.05, respectively). Sex, TNM stage, B7-H1, B7-H3, and T-cell intracellular antigen-1 expression remained significant prognostic factors after adjusting for other prognostic factors in a multivariate Cox proportional hazards regression model. In vitro studies revealed that knockdown of B7-H3 on tumor cells enhanced T-cell growth and interferon-γ secretion when stimulated by anti-CD3 and anti-CD28 monoclonal antibodies. Interferon-γ reduced CXCR4 expression on cancer cells and inhibited the CXCL12-induced cell migration. B7-H1 and B7-H3 are independent predictors of poorer survival in patients with NSCLC. Interference of the signal pathways of these negative regulatory molecules might be a new strategy for treating NSCLC.

## INTRODUCTION

Non-small cell lung cancer (NSCLC) accounts for 80%–85% of all lung cancer cases.[1] The majority of patients present with advanced disease. Although some improvements have been made, the five-year survival rate of NSCLC (16.6%) is still lower than many other leading cancers, such as the colon (64.2%), breast (89.2%) and prostate (99.2%) cancer. Recent studies suggested that some tumors have distinct molecular characteristics that allow them to be further classified into subsets of disease beyond the histologic level.[2] By focusing on cancer-specific molecular changes, targeted cancer therapies may be more effective than other traditional treatments, such as chemotherapy and radiotherapy, and less harmful to normal cells.[3]

Immune evasion is now recognized as a key feature of cancer progression.[4] Interactions among subsets of immune cells through costimulatory ligands and their receptors are essential for the initiation of an immune response. The lack of CD80 and CD86 expression on tumor cells is one of the mechanisms of the immune evasion of tumor cells.[5, 6] B7-H1 (PD-L1) and B7-H3, two novel members of B7/CD28 superfamily, complicate the immune response by adjusting the effects of costimulation on T-cells.[7, 8] B7-H1 is abundant on tumor cell lines and tumor tissues, and cancer cells expressing B7-H1 have been shown to increase apoptosis of antigen-specific human T-cell clones [4] and to inhibit CD4^+^ and CD8^+^ T-cell activation *in vitro*.[9] In addition, tumor cells transfected with B7-H1 were shown to grow even after adoptive T-cell immunotherapy, whereas blockade of PD-1/B7-H1 inhibited tumorigenesis *in vivo*.[10, 11] B7-H3, whose receptors have not yet been identified, is believed to be involved in both costimulatory and co-inhibitory pathways.[12] B7-H3 has been regarded as a positive regulation molecule as it was shown to stimulate CD4^+^ and CD8^+^T cells to increase the activity of cytotoxic T lymphocytes.[13] However, further studies revealed that B7-H3 could also inhibit the proliferation of T cells *in vitro* and reduce the secretion of interferon-γ (IFN-γ), tumor necrosis factor-α, granulocyte macrophage colony-stimulating factor, and other cytokines.

Prolonged survival has associated with a large number of tumor-infiltrating lymphocytes (TILs) in cancer, measured by the infiltration of CD3^+^ T cells [14], CD8^+^ T cells [15] or CD57^+^ NK cells.[16] Recent studies found that cytotoxic granule-associated RNA binding protein (TIA-1) was a good marker to detect cytotoxic cells, which coded for an integral membrane protein in cytotoxic granules, mainly in cytotoxic CD8^+^ T cells (CTLs, regardless of their activation state) and NK cells. [17] Tumor cells, which express a relatively restricted repertoire of chemokine and chemokine receptors, utilize and manipulate the chemokine system in a manner that benefits both local tumor growth and distant dissemination. Among the 19 chemokine receptors, CXCR4 is the receptor most widely expressed by malignant tumors and whose role in tumor biology is most thoroughly studied.[18] The functional expression of CXCR4 induces lung cancer cell migration and adhesion to stromal cells when binding to its unique ligand stromal cell-derived factor-1 (SDF-1), which in turn provides growth- and drug-resistance signals to tumor cells. CXCR4 antagonists, such as Plerixafor (AMD3100) and T140 analogues (TN14003/BKT140), can disrupt CXCR4-mediated tumor cell adhesion to stromal cells and sensitize lung cancer cells to cytotoxic drugs.[19] Study in head and neck squamous cell carcinoma showed that IFN-γ could also significantly reduce the expression of CXCR4.[20]

Although B7-H1 and B7-H3 was reported to express in cancer, their expression in NSCLC has not been fully characterized. Therefore, we sought to investigate their expression in NSCLC samples. In the present study, we evaluated B7-H1 and B7-H3 expression in NSCLC tissues via immunohistochemical analysis to determine the relationship between their expression and other clinicopathologic variables and their value in prognosis. We also assessed the association between B7-H1 and B7-H3 expression on tumor cells and TIA-1 and IFN-γ expression on TILs. The biological effects and their mechanisms of tumor-associated B7-H3 on T-cell proliferation and tumor cell migration were also explored.

## RESULTS

### B7-H1 and B7-H3 expression in NSCLC tissues

**Figure 1:**
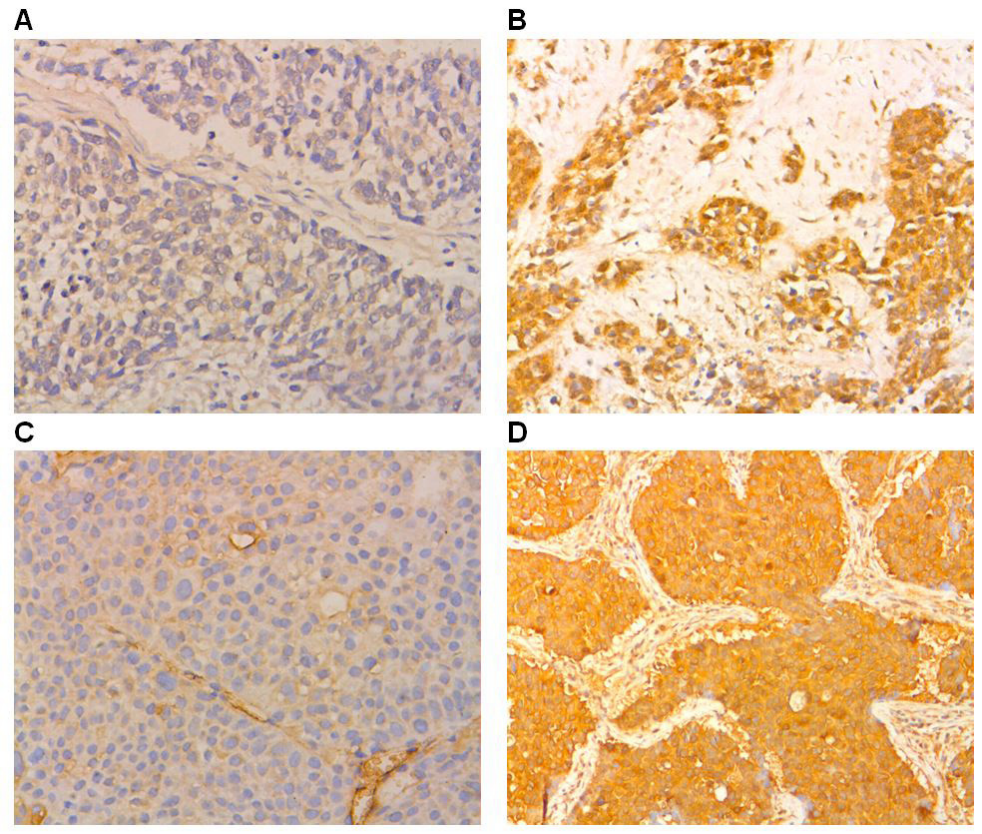
Immunohistochemical staining showing B7-H1 and B7-H3 expression in NSCLC and normal lung tissues (original magnification ×100) (A) Negative cytoplasmic expression of B7-H1 (B) Positive cytoplasmic expression of B7-H1 (C) Negative cytoplasmic expression of B7-H3 (D) Positive cytoplasmic expression of B7-H3.

Staining for B7-H1 was observed in the cell cytoplasm and membrane in both cancerous and noncancerous cells. However, staining for B7-H3 was only found in cancerous cells. B7-H1 was more commonly expressed in NSCLC tissues (93/128 cases, 72.7%) than in adjacent normal tissues (12/128 cases, 30%; p< 0.01; Figure 1). Similarly, B7-H3 was more commonly expressed in NSCLC tissues (89/128 cases, 69.5%) than in adjacent tissues (0/128 cases, 0%; p< 0.01; Figure 1).

### Relationship between B7-H1 and B7-H3 expression and clinicopathologic parameters

The relationship between tumor cell B7-H1/B7-H3 expression and clinicopathologic parameters was shown in (Table 1) B7-H1/B7-H3 expression in NSCLC tissue samples was associated with lymph node metastasis and advanced TNM stage (p<0.05 for both). Expression of either protein was not associated with sex, histopathologic type, or histologic grade. We also found a positive correlation between B7-H1 expression and B7-H3 expression in NSCLC tissue samples (p<0.05). Co-expression of B7-H1 and B7-H3 was observed in 68/128 (53.1%) NSCLC tissue samples but not in adjacent tissues. Furthermore, B7-H1 and B7-H3 co-expression was much more common in patients with lymph node metastasis (72.9%) than in those without lymph node metastasis (29.3%; p<0.01). The proportion of samples in which B7-H1 and B7-H3 were co-expressed increased as TNM stage (I, II, or III) increased (26.7%, 53.5%, and 82.5%, respectively; p<0.01). The co-expression of B7-H1 and B7-H3 was not significantly associated with age, sex, histopathologic type, histologic grade, and tumor size. (Table 1)

**Table 1:** Relationship between clinicopathologic parameters and B7-H1, B7-H3, TIA-1, IFN-γ, and CXCR4 expression in NSCLC (n=128)

Clinicopathologic parameters	No. of patients	B7-H1 expression		p value	B7-H3 expression		p value	TIA-1^+^ expression		p value	IFN-γ^+^ expression		p value	CXCR4 expression		p value
		Negative	Positive		Negative	Positive		Low	High		Low	High		Negative	Positive	
**Age at diagnosis (years)**																
≤60	51	17	34	**0.22**	10	41	0.03*	32	19	**0.06**	31	20	**0.53**	16	35	0.02*
>60	77	18	59		29	48		35	42		51	26		41	36	
**Sex**																
Male	91	21	70	**0.09**	30	61	**0.34**	42	49	0.03*	57	34	**0.60**	42	49	**0.56**
Female	37	14	23		9	28		25	12		25	12		15	22	
**Histopathologic type**																
Squamous cell carcinoma	61	16	45	**0.79**	23	38	**0.09**	26	35	0.04*	37	24	**0.45**	30	31	**0.31**
Adenocarcinoma	67	19	48		16	51		41	26		45	22		27	40	
**Differentiation**																
Poor	45	10	35	**0.34**	12	33	**0.73**	23	22	**0.49**	32	13	**0.25**	18	27	**0.54**
Moderate	22	7	15		6	16		14	8		13	9		10	12	
Well	61	18	43		21	40		30	31		37	24		29	32	
**Tumor size**																
≤3cm	38	13	25	0.04*	12	26	**0.59**	18	20	**0.34**	24	14	**0.93**	15	23	**0.63**
>3cm≤7cm	65	20	45		21	44		34	31		42	23		31	34	
>7cm	25	2	23		6	19		15	10		16	9		11	14	
**Lymph node metastasis**																
N_0_	58	21	37	0.04*	31	27	<0.01*	23	35	0.01*	31	27	0.02*	30	28	**0.14**
N_1_	70	14	56		8	62		44	26		51	19		27	43	
**TNM stage**																
I	45	15	30	0.04*	28	17	<0.01*	12	33	<0.01*	23	22	<0.01*	23	22	0.01*
II	43	15	28		8	35		20	23		25	18		26	17	
III	40	5	35		3	37		35	5		34	6		8	32	

* p<0.05

### Relationship between TIA-1, IFN-γ, and CXCR4 expression and clinicopathologic parameters

The rates of TIA-1^+^ and IFN-γ^+^ cell high infiltration (47.7% and 35.9%, respectively) in NSCLC tissues were significantly higher than those in adjacent tissues (p<0.01, respectively). The infiltration levels of TIA-1^+^ and IFN-γ^+^ cells in NSCLC tissues decreased in the presence of lymph node metastasis and as the TNM pathologic stage increased (p<0.05, respectively), but the rate of TIA-1^+^ cell high infiltration in squamous cell carcinomas (57.4%) was obviously higher than that in adenocarcinomas (38.8%; p=0.04). The overexpression (55.5%) of CXCR4 in NSCLC tissues was significantly higher than that in the adjacent tissues (p<0.01). CXCR4 was only minimally expressed in the adjacent tissues. CXCR4 expression increased as the TNM pathologic stage increased (p=0.01). (Table 1)

In NSCLC tissues, B7-H3 expression was positively correlated with CXCR4 expression (r=0.36; p<0.01). The high infiltration rate of TIA-1^+^ cells was positively associated with the high infiltration rate of IFN-γ^+^ cells (r=0.28; p<0.01).

### Relationship between B7-H1 and B7-H3 expression and OS

At the time of the final follow-up, 85 of 128 patients had died; of the remaining 43, 42 were alive and 1 was lost to follow-up. Patients whose tumors were positive for B7-H1 had shorter OS times than those whose tumors were negative for B7-H1 (28.7 months vs 60.6 months; p<0.01). Other prognostic factors were also shown to negatively affect OS, including B7-H3 (25.0 months vs 60.6 months; p<0.01) and CXCR4 expression (28.7 months vs 40.7 months; p= 0.08). Patients whose tumors were negative for both B7-H1 and B7-H3 had the best prognosis (60.6 months), whereas those with tumors in which both B7-H1 and B7-H3 were expressed had the poorest prognosis (22.7 months). NSCLC patients with high infiltration rates of TIA-1^+^ or IFN-γ^+^ cells had a better prognosis than those with low infiltration rates.(Table 2) After adjusting for sex, tumor differentiation, tumor size, lymph node metastasis, TNM stage, and expression of B7-H1, B7-H3, TIA-1^+^, IFN-γ^+^, and CXCR4, patients whose tumors were positive for B7-H1 (HR, 1.90; 95% CI, 1.09-3.30; p=0.02) or B7-H3 (HR, 2.26; 95% CI, 1.21-4.23; p=0.01) were at an increased risk of death. (Table 3) In addition, patients who were male, had advanced TNM stage disease, or had low infiltration of TIA-1^+^ in their tumors were also at an increased risk of death (p<0.05 for both).

**Table 2:** Correlation between molecular markers and median OS in NSCLC patients (univariate analysis)

Molecular markers	Cases (n)	MST (months)	Log-rank p value
**B7-H1**			
negative	35	60.6	<0.01*
positive	93	28.7	
**B7-H3**			
negative	39	60.6	<0.01*
positive	89	25.0	
**TIA-1^+^**			
low infiltration	67	20.5	<0.01*
high infiltration	61	59.2	
**IFN-γ^+^**			
low infiltration	82	28.7	0.02*
high infiltration	46	44.7	
**CXCR4**			
negative	57	40.7	0.08
positive	71	28.7	
**B7-H1/B7-H3**			
B7-H1−/B7-H3−	14	60.6	<0.01*
B7-H1−/B7-H3+	21	37.1	
B7-H1+/B7-H3−	25	49.5	
B7-H1+/B7-H3+	68	22.7	

* p<0.05; MST, median survival time

**Table 3:** Correlation between clinicopathologic parameters and median OS in NSCLC patients (multivariate analysis)

Clinicopathologic parameters	HR	95.0% CI	p value
**Sex**	0.52	0.30-0.90	0.02*
**Tumor differentiation**	0.74	0.52-1.06	0.10
**Tumor size**	0.85	0.63-1.16	0.31
**Lymph node metastasis**	0.92	0.42-2.01	0.84
**TNM stage**	1.82	1.05-3.16	0.03*
**B7-H1**	1.90	1.09-3.30	0.02*
**B7-H3**	2.26	1.21-4.23	0.01*
**TIA-1^+^**	0.32	0.16-0.63	<0.01*
**IFN-γ^+^**	1.45	0.78-2.67	0.24
**CXCR4**	0.86	0.53-1.38	0.52

* p<0.05; HR, hazard ratio; CI, confidence interval.

### B7-H3 expression on lung cancer cell lines and its association with metastasis

**Figure 2:**
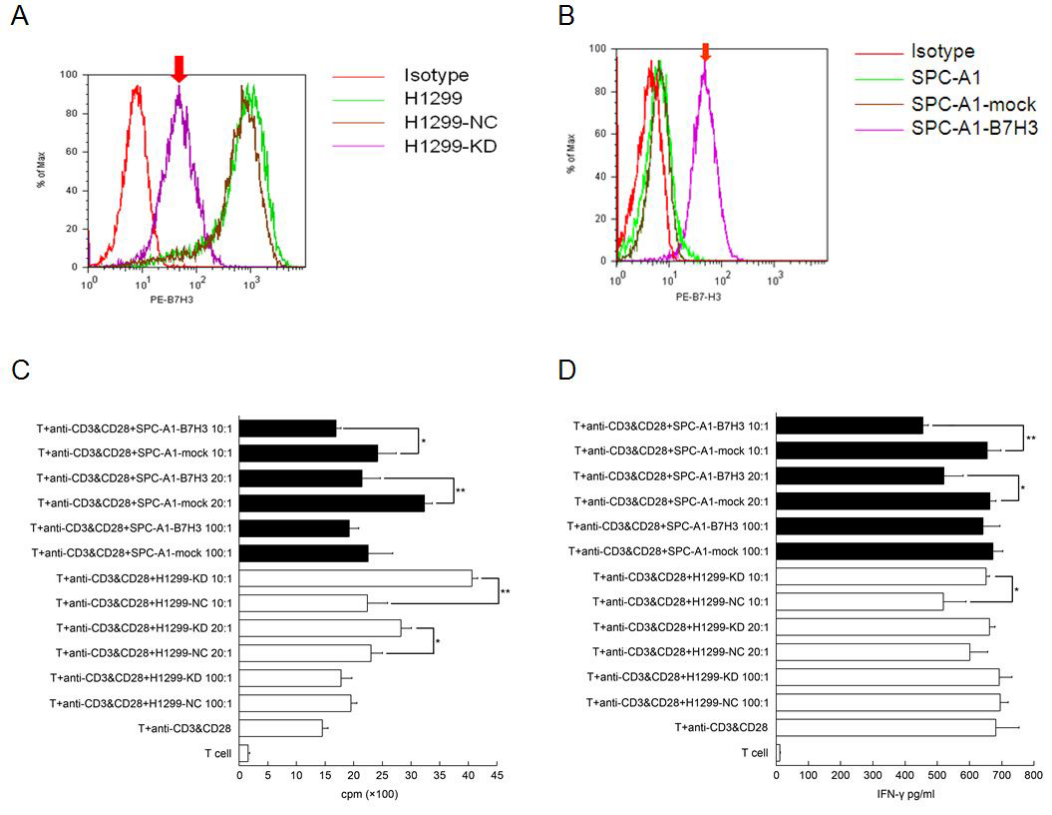
Effects of B7-H3 expression on T-cell proliferation and IFN-γ secretion (1) B7-H3 expression of on cell membrane of H1299 (A) and SPC-A1 (B) cells (2) T-cell proliferation (C) and IFN-γ secretion (D) stimulated by B7-H3 silencing or transfected cells (*p<0.05; **p<0.001) H1299-NC, vector-transfected H1299 cells; H1299-KD, B7-H3 knockdown H1299 cells; SPC-A1-mock, vector-transfected SPC-A1 cells; SPC-A1-B7-H3, B7-H3-transfected SPC-A1 cells. Experiments were done in triplicate and average ± SE displayed.

**Figure 3:**
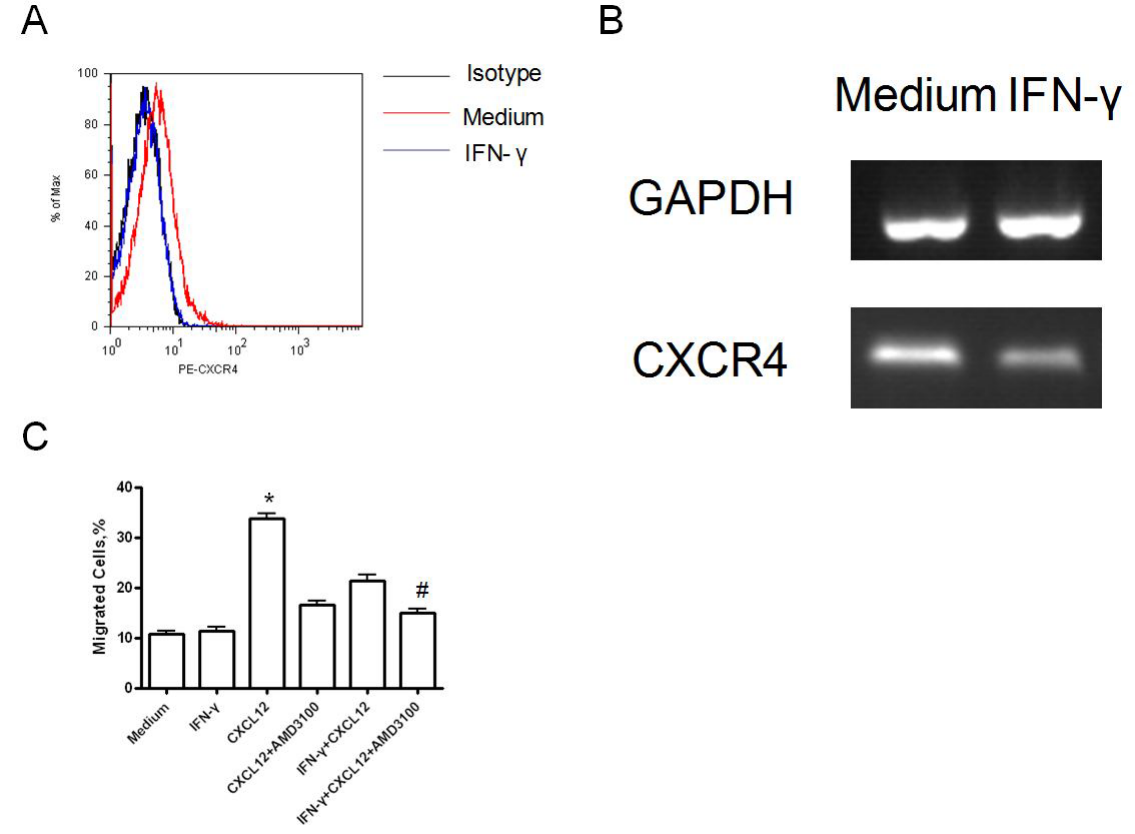
H1299 cell migration induced by IFN-γ after 72 hours (A) CXCR4 expression by flow cytometry; (B) CXCR4 expression by RT-PCR; (C) Migration ability of H1299 cells before and after IFN-γ stimulation (*p<0.001, compared with all other groups; ^#^p<0.01, compared with the IFN-γ+CXCL12 group) Experiments were done in triplicate and average ± SE displayed.

**Figure 4:**
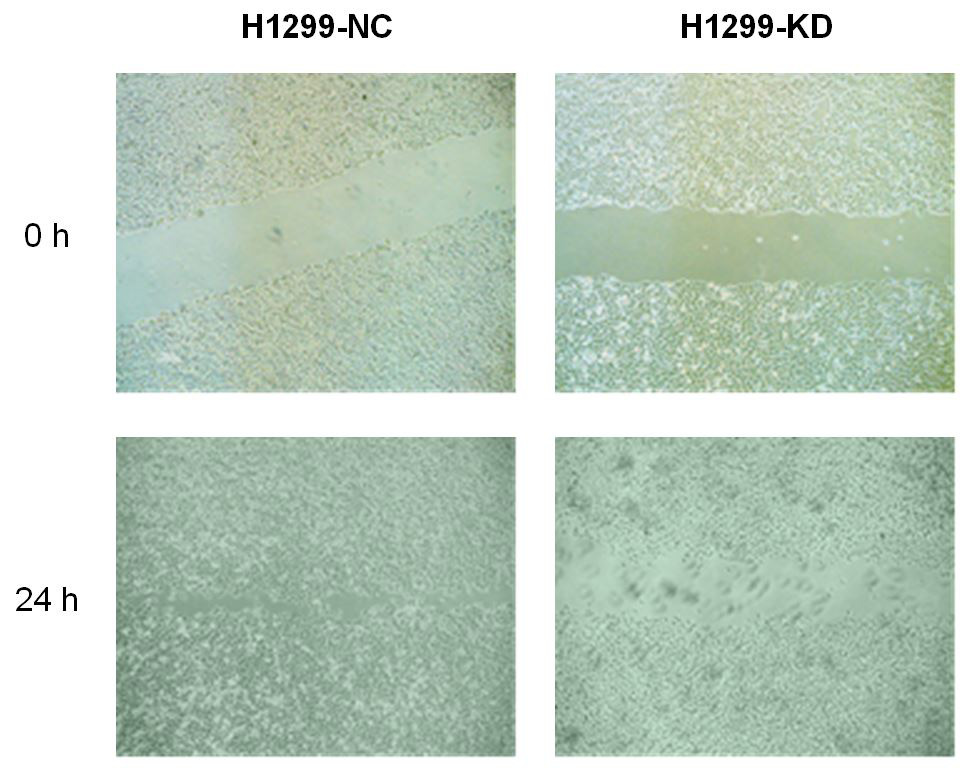
Migration capacity of H1299-KD and H1299-NC cells H1299-NC cells covered with scratches after 24 hours of incubation, whereas the same phenomena were not observed on H1299-KD cells H1299-NC, vector-transfected H1299 cells; H1299-KD, B7-H3 knockdown H1299 cells. Experiments were done in triplicate.

B7-H3 was highly expressed on lung cancer H1299 cells. The B7-H3 gene silencing on H1299 cells promoted the proliferation of T-cells and stimulated these cells to secrete IFN-γ with the costimulation by anti-CD3 mAb and anti-CD28 mAb *in vitro* (p<0.05). (Figure 2) It also showed that IFN-γ reduced CXCR4 expression on H1299 cells by both flow cytometry and RT¬-PCR. (Figure 3A and B) Like AMD3100 (a CXCR4 antagonist), IFN-γ significantly inhibited the migration capacity of H1299 cells stimulated by CXCL12 (p<0.01) (Figure 3C). *In vitro* scratch assay revealed that the knockdown of B7-H3 on H1299 cells slowed the migration of cells toward the scratch. (Figure 4) However, when B7-H3 gene was transfected to B7-H3 low expressed lung cancer SPC-A1 cells, we found a significantly inhibition of proliferation and IFN-γ secretion of T-cells (p<0.05). (Figure 2)

## DISCUSSION

Our results revealed cytoplasmic and membrane expression of B7-H1 and B7-H3 in surgically resected NSCLC specimens. The expression patterns of these molecules were consistent with those reported in previous studies that examined their expression in human tumor tissue.[4, 9, 10, 21] B7-H1 and B7-H3 were more likely to be expressed in NSCLC cells than in normal lung tissues, and B7-H1/B7-H3 expression was associated with decreased OS in patients with NSCLC. Thus, B7-H1/B7-H3 expression in NSCLC tissues could be a useful independent predictor of prognosis. Biological studies showed that B7-H3 was a negative regulatory molecule, as it inhibited T-cell proliferation and promoted tumor cell migration.

Accumulating evidence [22] has indicated that B7 family molecules are widely expressed in NSCLC and that their interactions with corresponding receptors are directly related to T-cell engagement of antigens. Previous studies revealed low expression of B7-H1 and high expression of B7-H3 in NSCLC. B7-H1 and B7-H3 expression was associated with a decreased number of TILs.[23-25] However, a recent study by Boland *et al*. did not reveal an association with B7-H1 or B7-H3 expression and the number of TILs in squamous cell lung carcinoma. Our study showed that B7-H1 and B7-H3 expression and the number of infiltrating TIA-1^+^ and IFN-γ^+^ cells in NSCLC tissues were significantly higher than those in the adjacent tissues. Statistical analysis revealed that high B7-H1 or B7-H3 expression was associated with lymph node metastasis (p<0.05) and TNM stage (p<0.05).

Only one study showed B7-H1 expression in NSCLC positivity correlated with survival shorter than 3 years after lobectomy[23, 26, 27], and the findings of studies of B7-H3 and survival were controversial. Xu *et al*. [24] reported that low B7-H3 expression was associated with poor prognosis in NSCLC, whereas, Bonald *et al*.[27] found no association between B7-H3 expression and poorer survival in squamous cell carcinoma of the lung. In this study, we found that B7-H1, B7-H3, and TIA-1 expression remained significant prognostic factors after adjusting for other prognostic factors in a multivariate Cox proportional hazards regression model. It showed that B7-H1 and B7-H3 could repress the antitumor immune response in NSCLC by inhibiting the infiltration of cells that express TIA-1 and IFN-γ. Thus, our results indicate that B7-H1 and B7-H3 expression is an independent predictor of poor prognosis in patients with NSCLC. The interference of the signal pathways of these negative regulatory molecules might be a new strategy for treating NSCLC. Our results indicate that B7-H3 has inhibitory effects on the immune system; however, the real function of B7-H3 in human NSCLC could be more complicated.

The mechanisms regulating B7-H3 expression on tumor cells are not well known. Previous studies suggested that the cytokine microenvironment induced the expression of B7-H1 on tumor cells.[23, 28] In addition, T-cells or natural killer cells that infiltrated into tumor tissue were shown to secrete IFN-γ and some other cytokines. In the current study, decreasing IFN-γ prompted CXCR4 expression on cancer cells, which could help tumor cells escape from immunity. These results suggest that B7-H3 may be involved in the development of human NSCLC.

One limitation of our study was the relatively small sample, which could lead to an overestimation of the magnitude of relationships between variables. Our patients had stage I-III disease and received various kinds of therapy, which may have introduced treatment bias. Nonetheless, our findings suggest that B7-H1 and B7-H3 play an important role in tumor metastasis and thus could be applied as prognostic markers and/or targets for NSCLC therapy. Further investigations are necessary to clarify and understand the role of B7-H1 and B7-H3 in patients with NSCLC.

## MATERIALS AND METHODS

### Tissue samples

Primary tumor samples and clinical data were obtained from 128 patients with NSCLC who underwent surgery without any preoperative therapy (91 men and 37 women; median age at diagnosis, 62.5 years) between March 2005 and October 2007 at the First Affiliated Hospital of Soochow University and Shanghai Renji Hospital in China. NSCLC stage was classified according to the AJCC tumor-node-metastasis (TNM) staging system (2002 version).[29] Cell differentiation was determined using the World Health Organization (WHO) classification (2000 revision).[30] Data on patients' age, sex, tumor location, histopathologic type, histologic grade, tumor size, lymph node invasion, and tumor stage were extracted from medical records. The median follow-up time was 53.3 months (range, 40.3-74.0 months), and the most recent follow-up correspondence was initiated on March 31, 2011. The study was conducted after informed consent was obtained from all patients and approval from an independent research ethics committee.

### Cell lines

Lung cancer cell lines H1299 (HLA-A*0201^−^) and SPC-A1 (HLA-A*0201^−^) were form Institute of Health Sciences, Shanghai Institutes for Biological Sciences, Chinese Academy of Sciences and maintained in RPMI-1640 medium supplemented with 10% fetal calf serum.

### Immunohistochemical analysis

Immunostaining was performed using the EliVision plus kit (Maixin-Bio, Fuzhou, China). Formalin-fixed, paraffin-embedded tissue blocks were sectioned to 3 μm and mounted on charged glass slides (Fisher Scientific, Pittsburgh, PA). Antigen retrieval was done in a citrate buffer [20 mmol/L (pH 6.0)] at 120°C for 10 minutes. Endogenous peroxidase activity was blocked with 3.0% hydrogen peroxide for 10 minutes. Mouse anti-human B7-H1 monoclonal antibody (mAb; diluted 1:100; Clone 2H11) [31] and mouse anti-human B7-H3 mAb (diluted 1:100; Clone 4H7) [32] were used as the primary antibodies, and mouse immunoglobulin G was used as the negative control. Mouse anti-human TIA-1 mAb, mouse anti-human IFN-γ mAb, and mouse anti-human CXCR4 mAb were purchased from Santa Cruz Biotechnology (Santa Cruz, CA) and used according to the manufacturer's instructions. For visualization, the sections were incubated with 3, 3-diaminobenzidine solution and counterstained with hematoxylin.

### Evaluation of immunostaining

Histologic analysis was performed by two investigators simultaneously without knowledge of the patients' clinical characteristics. B7-H1 and B7-H3 expression was measured as the percentage of tumor cells displaying immunoreactivity in the cytoplasm or on the membrane, which was determined by counting the number of B7-H1- or B7-H3-stained tumor cells out of 1,000 tumor cells in each section. Cells were counted at 400× magnification in at least 10 fields in randomly selected tumor areas. Staining was evaluated using a semiquantitative assay previously described by Remmele *et al*.[33] The immunoreactive score was calculated by multiplying the staining intensity. The percentage of cells with positive staining was scored as follows: 0 = no staining, 1 = weak staining, 2 = moderate staining, and 3 = strong staining. The percentage of positively stained cells was scored as follows: 0 = no staining, 1 = 1%-10% of cells, 2 = 11%-50% of cells, and 3 = more than 50% of cells stained. The total score per sample therefore ranged from 0 to 9 whereby 0 indicated no staining (e.g., negative results), 1 indicated weak staining, 2 or 3 indicated moderate staining, and more than 3 indicated strong staining. In the final analysis, samples with no staining or weak staining were considered to have negative expression, and samples with moderate or strong staining were considered to have positive expression.

Whole areas of each section were surveyed microscopically at 40× magnification, and cells counts were performed at 400× magnification in at least five fields in randomly selected tumor areas. The density of tumoral lymphoid infiltrates was quantified by counting the small round lymphocytes distributed within the tumor epithelium and the peritumoral stroma. TIA-1-expressing TILs were scored as follows: 0 = no staining, 1 = 1-25 cells stained, 2 = 26-50 cells stained, and 3 = 51 or more cells stained. IFN-γ-expressing TILs were scored as follows: 0 = no staining, 1 = 1-5 cells stained, 2 = 6-19 cells stained, and 3 = 20 or more cells stained. Scores from 0 to 1 were considered low infiltration, whereas scores from 2 to 3 were defined as high infiltration.[34]

### Reverse transcriptase polymerase chain reaction

The tumor cells were harvested and lysed in TRIzol reagent (Invitrogen, CA). First-strand cDNA was prepared using random primers and following the manufacturer's instructions for the SuperScript III First-Strand Synthesis kit (Invitrogen, CA). Synthesis of cDNA was controlled by performing reverse transcriptase polymerase chain reaction (RT-PCR) using glyceraldehyde 3-phosphate dehydrogenase primers. RT-PCR with the primers 5′-TACTCGAAGCCCAGCATGACC-3′ (forward primer) and 5′-CCACCAGCAGTGCAATGAGAC-3′ (reverse primer) specific for human B7-H3, the primers 5′-CTAATTATTCGGTAACTGACTTGA-3′ (forward primer) and 5′-ACAGTTCAGCCATCACTTGGA-3′ (reverse primer) specific for human IFN-γ, and the primers 5′-GTCGTGGAGTCTACTGGCGTCTT-3′ (forward primer), 5′-CAGTCTTCTGAGTGGCAGTGATGG-3′ (reverse primer) specific for human glyceraldehyde 3-phosphate dehydrogenase was performed using TaKaRa LA Taq enzyme (Takara, Shiga, Japan). Cycle conditions were 94°C for 30 seconds, 58°C for 30 seconds, and 72°C for 30 seconds for 35 cycles.

### Flow cytometry

Cell surface expression was determined via immunofluorescence staining and flow cytometry with the Coulter Epics XL flow cytometer with EXPO32 software (Beckman Coulter, CA). The cells were incubated with anti-B7-H1 mAb (2H11) or anti-B7-H3 mAb (4H7) for 30 minutes at room temperature. After being washed thoroughly with phosphate-buffered saline (PBS), the cells were stained with phycoerythrin-conjugated goat anti-mouse antibodies (Immunotech, Marseille, France) for 30 minutes at 4°C. Non-specific staining was determined using isotype control antibodies.

### Transfection

SPC-A1 cells were transfected with the pIRES2-EGFP plasmid containing the full-length human B7-H3 gene (SPC-A1-B7-H3) or with the wild-type pIRES2-EGFP plasmid (SPC-A1-Mock) and selected on the basis of G418 resistance. B7-H3 knockdown (H1299-KD) and vector control (H1299-NC) H1299 cells were transfected with the corresponding pGPU6/GFP/Neo plasmids (GenePharma, Shanghai, China), and the expression of surface molecules was confirmed by flow cytometry using an anti-B7-H3 mAb (4H7).

### *In vitro* analysis of T-cell responses

For T-cell proliferation assays, 96-well flat-bottom plates were coated with 1 μg/ml agonist anti-CD3 and 5 μg/ml anti-CD28 mAb (Immunotech, Marseille, France) in PBS at 4°C overnight. Either mitomycin-treated tumor cells or medium alone was added to the plates cocultured with 1×10^5^ purified T-cells obtained from peripheral blood mononuclear cells (HLA-A*0201^−^) at an effector-to-target ratio of 1:10, 1:20, and 1:100. Then, T-cells were incubated for 72 hours and pulsed with 1 μCi of [^3^H] thymidine. Sandwich enzyme-linked immunosorbent assay for human IFN-γ was performed according to the manufacturer's instructions (R&D Systems, Minneapolis, MN).

### Migration assays

Transwell migration assays were conducted on H1299 cells with natural B7-H3 gene expression. Tumor cells were pretreated with IFN-γ (10 ng/ml) or AMD3100 (10 μg/ml) for 24 hours. Transwell chambers (8-μM pore) were coated with 10 μg/mL fibronectin (Sigma-Aldrich, St. Louis, MO) and incubated overnight at 4°C, washed in PBS, and rehydrated with serum-free media for 30 minutes at 37°C. The media were removed, and cells (1×10^5^/chamber) were added to upper chambers with 100 ng/ml CXCL12 in lower chambers as a chemoattractant. IFN-γ was chosen as a control. The chambers were incubated for 20 hours at 37°C and 5% CO_2_. Migrated cells on the underside of the filter were fixed in 10% ethanol for 20 minutes prior to staining with 4,6-diamidino-2-phenylindole (Sigma-Aldrich, St. Louis, MO) for 10 minutes at room temperature. Membranes were mounted on glass slides, and cells were enumerated using flow cytometry (Beckman Coulter, Brea, CA) to analyze multiple fluorescent micrographs. Results from three independent experiments with three replicates per experiment were pooled.

Scratch-wound migration assays were conducted on H1299 cells after B7-H3 gene knockdown. When cell monolayers reached 90% confluence, a scratch wound was made using a pipette tip. The closure of the wound was measured after 20 hours.

### Statistical analysis

Comparisons between unpaired groups were made using the Mann-Whitney *U* test. Correlations between the parameters were evaluated with Spearman's rank correlation test. The overall survival (OS) times of patients whose specimens did or did not have B7-H1/B7-H3 expression were compared using the Kaplan-Meier method of survival analysis and the log-rank test. For all patients, survival time was calculated from the date of pathologic diagnosis of cancer to the date of death or last follow-up. The data from patients who were alive at the last day of follow-up were censored. The median follow-up time was calculated using only censored data. The median survival time was also calculated. Hazard ratios (HRs) and 95% confidence intervals (CIs) were estimated using univariate and multivariate Cox proportional hazard models. All statistical testing was conducted using SPSS software, version 20.0 (Chicago, IL). p values were two-sided, and p<0.05 was considered statistically significant.
